# CYP79D enzymes contribute to jasmonic acid-induced formation of aldoximes and other nitrogenous volatiles in two *Erythroxylum* species

**DOI:** 10.1186/s12870-016-0910-5

**Published:** 2016-10-04

**Authors:** Katrin Luck, Jan Jirschitzka, Sandra Irmisch, Meret Huber, Jonathan Gershenzon, Tobias G. Köllner

**Affiliations:** 1 Max Planck Institute for Chemical Ecology, Hans-Knöll-Strasse 8, D-07745 Jena, Germany; 2 Present address: Fraunhofer Institute for Molecular Biology and Applied Ecology IME, Forckenbeckstrasse 6, D-52074 Aachen, Germany; 3 Present address: Michael Smith Laboratories, University of British Columbia, Vancouver, Canada

**Keywords:** *Erythroxylum*, Cytochrome P450 monooxygenase, CYP79, Aldoxime, Volatiles

## Abstract

**Background:**

Amino acid-derived aldoximes and nitriles play important roles in plant defence. They are well-known as precursors for constitutive defence compounds such as cyanogenic glucosides and glucosinolates, but are also released as volatiles after insect feeding. Cytochrome P450 monooxygenases (CYP) of the CYP79 family catalyze the formation of aldoximes from the corresponding amino acids. However, the majority of CYP79s characterized so far are involved in cyanogenic glucoside or glucosinolate biosynthesis and only a few have been reported to be responsible for nitrogenous volatile production.

**Results:**

In this study we analysed and compared the jasmonic acid-induced volatile blends of two *Erythroxylum* species, the cultivated South American crop species *E. coca* and the African wild species *E. fischeri*. Both species produced different nitrogenous compounds including aliphatic aldoximes and an aromatic nitrile. Four isolated *CYP79* genes (two from each species) were heterologously expressed in yeast and biochemically characterized. CYP79D62 from *E. coca* and CYP79D61 and CYP79D60 from *E. fischeri* showed broad substrate specificity *in vitro* and converted L-phenylalanine, L-isoleucine, L-leucine, L-tryptophan, and L-tyrosine into the respective aldoximes. In contrast, recombinant CYP79D63 from *E. coca* exclusively accepted L-tryptophan as substrate. Quantitative real-time PCR revealed that *CYP79D60*, *CYP79D61*, and *CYP79D62* were significantly upregulated in jasmonic acid-treated *Erythroxylum* leaves.

**Conclusions:**

The kinetic parameters of the enzymes expressed *in vitro* coupled with the expression patterns of the corresponding genes and the accumulation and emission of (*E/Z*)-phenylacetaldoxime, (*E/Z*)-indole-3-acetaldoxime, (*E/Z*)-3-methylbutyraldoxime, and (*E*/*Z*)-2-methylbutyraldoxime in jasmonic acid-treated leaves suggest that CYP79D60, CYP79D61, and CYP79D62 accept L-phenylalanine, L-leucine, L-isoleucine, and L-tryptophan as substrates *in vivo* and contribute to the production of volatile and semi-volatile nitrogenous defence compounds in *E. coca* and *E. fischeri*.

**Electronic supplementary material:**

The online version of this article (doi:10.1186/s12870-016-0910-5) contains supplementary material, which is available to authorized users.

## Background

Plant volatiles play diverse roles in the interactions between plants and their environment. Flower volatiles, for example, can attract pollinators while vegetative volatiles are involved in plant defence, either directly by repelling the attacker or indirectly by e.g. attracting herbivore enemies [1–5]. The formation and emission of vegetative volatiles is often induced by chewing or sucking herbivores and the resulting volatile blends usually contain dozens of substances from diverse classes of natural compounds [6–9]. Herbivore-induced volatile blends are in general dominated by terpenes and green leaf volatiles (GLVs, C_6_ aldehydes, alcohols and esters derived from fatty acid cleavage), but comprise also aromatic compounds, alcohols, and nitrogen-containing amino acid derivatives [10]. While the formation and biological roles of terpenes and GLVs have been extensively studied in the past, our knowledge about the other components of herbivore-induced volatile blends is still limited.

Nitrogen-containing vegetative volatiles such as aldoximes, nitriles and nitro compounds are widely distributed among the angiosperms and have been reported from e.g. the Salicaceae, Fabaceae, Solanaceae, Cucurbitaceae, Rutaceae, Rosaceae, and Poaceae [11]. Poplars (Salicaceae), for example, release a complex mixture of aliphatic aldoximes, aliphatic and aromatic nitriles, and an aromatic nitro compound in response to herbivory by gypsy moth (*Lymantria dispar*) larvae [7, 11, 12]. Although these nitrogen-containing volatiles are minor components of the total blend, they likely play important roles in indirect and direct poplar defence. Electrophysiological recordings and olfactometer bioassays revealed that volatile aldoximes were more attractive for a gypsy moth parasitoid than the major terpenes and GLVs [12]. Moreover, poplar nitriles were shown to be repellent for gypsy moth caterpillars, while volatile and semi-volatile aldoximes had toxic effects on these larvae [11, 13].

Aldoximes and nitriles are produced from amino acids through the action of cytochrome P450 monooxygenases (CYP) of the CYP79 and CYP71/736 families (recently reviewed in [14]). CYP79 enzymes accept amino acids as substrates and catalyse the formation of aldoximes by two successive *N*-hydroxylations, a dehydration and a decarboxylation reaction [15, 16]. The aldoximes formed can then serve as substrates for CYP71 enzymes, which convert them into the corresponding nitriles [13]. The first characterized CYP79 enzyme, CYP79A1 from *Sorghum bicolor*, was identified and characterized in 1995 by Sibbesen and co-workers [16]. It catalyses the reaction from L-tyrosine to *p*-hydroxyphenylacetaldoxime, which is further converted into the cyanogenic glucoside dhurrin in sorghum [16]. While most of the CYP79 enzymes characterized so far produce aldoximes as precursors for cyanogenic glucosides, glucosinolates, and other non-volatile nitrogen-containing defence compounds, a few CYP79s from two different poplar species (CYP79D6v3 and CYP79D7v2 from *Populus trichocarpa* and CYP79D6v4 from *P. nigra*) have been reported to be responsible for herbivore-induced volatile production [11, 12, 17]. CYP79s involved in cyanogenic glucoside and glucosinolate formation usually possess high substrate specificity, thus determining the specificity of the entire pathway [18–21]. In contrast, poplar CYP79D6 and CYP79D7 have broader substrate specificity and produce complex mixtures of volatile and semi-volatile aldoximes [11, 12, 17].

To expand our knowledge about the formation of volatile aldoximes and nitriles, we have now begun to investigate and compare their biosynthesis in the genus *Erythroxylum.* Two species with different geographical origins and cultivation histories were chosen for this analysis*. Erythroxylum coca* is an economically and pharmacologically important crop cultivated on the eastern slopes of the Andes since more than 8000 years. *E. fischerii*, in contrast, is a wild species native to the tropical forests in Africa. Both species are members of the Erythroxylaceae, which belong, like poplars, to the diverse order Malpighiales. Since it has been shown that the formation of volatiles can be induced by artificial treatments with the plant hormone jasmonic acid (JA) (e.g. [12, 22]), we measured and compared volatile emission in response to JA treatment in *E. coca* and *E. fischeri* and detected numerous nitrogen-containing compounds. Candidate *CYP79* genes isolated from both species were then heterologously expressed in yeast, and enzyme characterization and gene expression analysis indicated a potential function of individual *Erythroxylum* CYP79 proteins in volatile aldoxime formation.

## Results

### Jasmonic acid induces the emission of nitrogenous volatiles in *Erythroxylum coca* and *E. fischeri*

Many plant species respond to herbivory with an increased JA accumulation that induces the biosynthesis of diverse plant defence compounds including nitrogen-containing volatiles [23]. Hence to study the formation of nitrogenous volatiles in *Erythroxylum* species, we collected and compared the volatile blends of untreated and JA-treated twigs of *E. coca* and *E. fischeri*. Although both species emitted volatiles from untreated twigs, JA-treatment significantly increased volatile emission (Table 1). The blends from control and JA-treated twigs of *E. coca* and *E. fischeri* were dominated by monoterpenes (e.g. (*E*)-β-ocimene, mentha-1,5,8-triene, and linalool), sesquiterpenes (e.g. β-elemene and (*E,E*)-α-farnesene) and the homoterpenes (3*E*)-4,8-dimethylnona-1,3,7-triene (DMNT) and (3*E*,7*E*)-4,8,12-trimethyltrideca-1,3,7,11-tetraene (TMTT). In addition, both species produced significant amounts of nitrogenous volatiles such as (*E/Z*)-2-methylbutyraldoxime, (*E/Z*)-3-methylbutyraldoxime, benzyl cyanide, phenylnitroethane, an unidentified nitro compound, and indole in response to JA treatment (Table 1). As typical herbivore-induced vegetative volatiles, green leaf volatiles were also present in JA-induced *E. coca* and *E. fischeri* blends. Notably, the qualitative compositions of the JA-induced volatile blends of both species were nearly identical and the total amounts of released volatiles were in the same range. However, there were major quantitative differences in the emission of single volatiles between *E. coca* and *E. fischeri* (Table 1). While *E. coca*, for instance, emitted β-elemene as major sesquiterpene and produced minor amounts of (*E,E*)-α-farnesene, *E. fischerii* released large amounts of (*E,E*)-α-farnesene and produced only traces of β-elemene. Another remarkable difference was found for indole, which was one of the dominant nitrogen-containing volatiles in *E. coca* but was a minor compound in *E. fischeri*.

**Table 1:** Volatile compounds of *Erythroxylum coca* and *E. fischeri* released from untreated twigs (control) and jasmonic acid-treated twigs (JA treatment)

	*Erythroxylum coca*			*Erythroxylum fischeri*		
Compound	Control	JA treatment	*P*-value	Control	JA treatment	*P*-value
	(Mean ± SE)	(Mean ± SE)		(Mean ± SE)	(Mean ± SE)	
N-containing volatiles						
(*E*)-3-methylbutyraldoxime*	0.6 ± 0.4	40.1 ± 9.6	**0.020**	0 + 0	23.7 + 7.4	**0.037**
(*E*)-2-methylbutyraldoxime*	0.4 ± 0.4	51.5 ± 9.7	**0.018**	0 + 0	16.1 + 10.6	**0.037**
(*Z*)-2-methylbutyraldoxime*	0.2 ± 0.2	9.5 ± 2.2	**0.018**	0 + 0	4.4 + 1.3	**0.037**
(Z)-3-methylbutyraldoxime*	0 ± 0	29.7 ± 6.6	**0.014**	0 + 0	17.1 + 7.2	**0.037**
benzyl cyanide*	0.1 ± 0.1	315.6 ± 76.5	**0.018**	2.1 + 1.2	241 + 199.7	**0.050**
phenylnitroethane	3.7 ± 0.7	99 ± 26.4	**0.021**	7.7 + 4.6	62.4 + 39.8	**0.050**
unidentified nitro compound	0.1 ± 0.1	3.1 ± 0.9	**0.018**	0.8 + 0.8	21 + 11.8	**0.046**
indole*	4.5 ± 2	256.2 ± 50.3	**0.021**	2.1 + 1	13.2 + 2.3	**0.050**
monoterpenoids						
myrcene*	4.7 ± 0.8	29.2 ± 3.5	**0.021**	5.7 + 1.8	36.1 + 4.7	**0.050**
(*Z*)-ocimene*	4.2 ± 0.5	107.7 ± 19.9	**0.021**	4.2 + 1.5	83.4 + 9.8	**0.050**
(*E*)-ocimene*	289.1 ± 54.1	14685.7 ± 4017.2	**0.021**	281.3 + 105.8	9257 + 847.3	**0.050**
allo-ocimene	0 ± 0	15.6 ± 2	**0.014**	0 + 0	1.7 + 0.4	0.037
mentha-1,5,8-triene	1.1 ± 0.2	25.6 ± 3.3	**0.021**	10.7 + 6.2	572.7 + 252.6	0.050
(*Z*)-linalool oxide	6.7 ± 1.6	130.7 ± 30.8	**0.021**	2.6 + 0.9	16.8 + 3	0.050
(*E*)-epoxy-ocimene	3.5 ± 0.6	780.2 ± 152.6	**0.021**	1.4 + 0.4	105.6 + 43.6	0.050
linalool*	64.4 ± 20.1	1709.8 ± 555.1	**0.021**	56 + 23.3	178.6 + 16.8	0.050
unidentified monoterpene 1	0 ± 0	6.5 ± 0.7	**0.014**	0.9 + 0.4	19.8 + 6.6	0.050
unidentified monoterpene oxide 1	0.1 ± 0.1	9 ± 1.9	**0.018**	0 + 0	1.9 + 1.4	0.121
unidentified monoterpene oxide 2	0.3 ± 0.2	11.5 ± 1.9	**0.020**	0 + 0	6 + 0.3	**0.037**
sesquiterpenoids						
β-elemene*	133.6 ± 32.4	208.2 ± 48.7	0.248	0 + 0	1 + 0	**0.037**
(*E*)-β-caryophyllene*	79.4 ± 17.1	72.9 ± 9.8	0.564	0.2 + 0.2	5.3 + 0.3	**0.046**
(*Z,E*)-α-farnesene	1.8 ± 1.8	16.2 ± 2.6	**0.018**	0 + 0	3.1 + 1.5	**0.037**
(*E,E*)-α-farnesene	17.5 ± 5.2	76.5 ± 14.6	**0.021**	240.4 + 176.9	1505.1 + 698.4	0.127
unidentified sesquiterpene 1	10.7 ± 4.9	26.6 ± 3.8	0.083	0.6 + 0.3	6.4 + 0.6	**0.050**
unidentified sesquiterpene 2	9.8 ± 4.4	20.9 ± 2.8	0.083	0.1 + 0.1	3.3 + 0.3	**0.046**
unidentified sesquiterpene 3	13.6 ± 5.8	24.7 ± 3.7	0.083	0 + 0	0.3 + 0.3	0.317
unidentified sesquiterpene 4	12.8 ± 7.5	46.5 ± 13.5	0.083	8.8 + 6.3	42.2 + 14.9	0.127
unidentified sesquiterpene 5	4.1 ± 2.4	19.8 ± 4.5	**0.042**	0.5 + 0.3	1.4 + 0.2	**0.050**
unidentified sesquiterpene 6	12 ± 3.3	23.6 ± 4	**0.043**	0 + 0	0.6 + 0.6	0.317
homoterpenes						
DMNT*	91.2 ± 20.2	112.1 ± 25.7	0.773	14.8 + 6.3	51.6 + 25.4	0.127
TMTT*	524.6 ± 138.9	1009.9 ± 218.1	**0.043**	187.6 + 91.2	630.5 + 159.4	**0.050**
diterpenes						
geranyl linalool*	3.8 ± 1	19.2 ± 3.9	**0.021**	0 + 0	0 + 0	NA
GLVs and ester						
(*Z*)-3-hexenyl acetate*	9.5 ± 3.8	15.7 ± 2.6	0.149	4.6 + 2.3	80.5 + 32.6	**0.050**
(*E*)-2-hexenyl acetate	0 ± 0	0 ± 0	NA	4.6 + 1	21.3 + 9	0.275
(Z)-3-hexenol*	0.1 ± 0.1	0 ± 0	0.317	0 + 0	11.3 + 0.3	**0.037**
(Z)-3-hexenyl propionate	13.1 ± 3.1	81.5 ± 14.8	**0.021**	1.9 + 0.6	14.8 + 7.9	**0.050**
(Z)-3-hexenyl isobutyrate	2.3 ± 0.2	91.5 ± 17.5	**0.014**	2.5 + 0.5	55 + 14.2	**0.037**
(Z)-3-hexenyl 2-methylbutanoate	0 ± 0	65.2 ± 24.7	**0.047**	0 + 0	8.2 + 4.5	0.121
(*E*)-3-hexenyl hexanoate	2.2 ± 0.5	36.7 ± 5.7	**0.021**	0.7 + 0.2	4.7 + 0.9	**0.050**
(*Z*)-3-hexenyl tiglate	35.4 ± 8.2	132.3 ± 18.1	**0.021**	1.2 + 0.3	6.2 + 1.5	**0.050**
unidentified ester	0 ± 0	6.1 ± 1	**0.021**	0 + 0	1.8 + 0.2	**0.050**
alcohols						
benzyl alcohol*	4.9 ± 1.4	53.1 ± 7	**0.021**	0.1 + 0.1	3.4 + 3.1	0.246
2-phenylethanol*	1.3 ± 0.3	104.3 ± 24.2	**0.021**	1 + 0.8	36.4 + 33.9	0.127
unidentified compounds						
unidentified compound 1	3.6 ± 0.7	39.5 ± 4.5	**0.021**	3.1 + 1.1	40.2 + 5.5	**0.050**
unidentified compound 2	0 ± 0	24 ± 4.6	**0.014**	0 + 0	5.5 + 2.1	**0.037**
unidentified compound 3	3.8 ± 0.6	35.7 ± 6.8	**0.021**	2.6 + 0.8	32.9 + 12.7	**0.050**
unidentified compound 4	0.1 ± 0.1	16.7 ± 3.2	**0.018**	0 + 0	14.7 + 5.9	**0.037**
unidentified compound 5	1.4 ± 0.9	31.4 ± 9.7	**0.020**	0 + 0	0 + 0	NA
unidentified compound 6	1.3 ± 1.3	85 ± 22.8	**0.018**	0 + 0	1.8 + 0.3	**0.037**
unidentified compound 7	56.4 ± 12.5	71 ± 7.2	0.386	21 + 10.7	41 + 12.1	0.275
unidentified compound 8	0 ± 0	12.9 ± 2.3	**0.014**	0 + 0	2.9 + 0.7	**0.037**
unidentified compound 9	0.5 ± 0.2	10.1 ± 1.1	**0.021**	0.5 + 0.3	9.7 + 1.7	**0.050**
unidentified compound 10	0.5 ± 0.2	18.8 ± 0.9	**0.021**	0.6 + 0.4	9.5 + 0.3	**0.050**
unidentified compound 11	1.9 ± 0.3	13.8 ± 0.6	**0.021**	0.6 + 0.2	7.2 + 0.8	**0.050**
unidentified compound 12	0 ± 0	10.7 ± 0.4	**0.014**	0 + 0	5.2 + 0.5	**0.037**
unidentified compound 13	0 ± 0	27.6 ± 5.6	**0.014**	0 + 0	7.1 + 7.1	0.317
**total volatiles**	**1508 ± 322**	**21101 ± 4605**	**0.021**	**874 ± 402**	**13351 ± 458**	**0.050**

Emission rates are displayed as means ± SE in ng g^-1^ fresh weight h^-1^ (*E. coca*, *n* = 4; *E. fischeri*, *n* = 3). *P*-values are based on the results from Kruskal-Wallis rank sum tests between the control and the JA-treatment. *P*-values ≤ 0.05 indicate significant differences and are shown in bold. Compounds identified using authentic standards are marked with asterisks (*). Unmarked compounds were identified by comparison of their mass spectra with those of reference libraries

### Identification of CYP79 enzymes from *E. coca* and *E. fischeri*

To identify putative *Erythroxylum CYP79* genes, a TBLASTN search against an in-house 454 cDNA sequencing database of *E. coca* young leaf tissue [24, 25] was conducted using the amino acid sequence of CYP79D6v3 from *Populus trichocarpa* [11] as input sequence. One sequence representing a putative P450 enzyme of the CYP79 family was identified. Amplification of this gene resulted in two highly homologous sequences that were designated as *CYP79D62* and *CYP79D63* according to the general P450 nomenclature (D.R. Nelson, P450 Nomenclature Committee). PCR with cDNA made from JA-treated *E. fischeri* leaves using the primer pair designed for amplification of *E. coca* sequences revealed an additional gene (*CYP79D60*). To identify further potential *CYP79D* candidates, primers specific to conserved regions among the obtained genes were designed and PCR was performed with cDNA made from JA-treated *Erythroxylum* leaves. While most of the resulting amplicons were identical to *CYP79D62, CYP79D63,* and *CYP79D60*, one fragment amplified from *E. fischeri* cDNA showed sequence divergence and the isolated full-length clone was designated as *CYP79D61*.

**Fig. 1:**
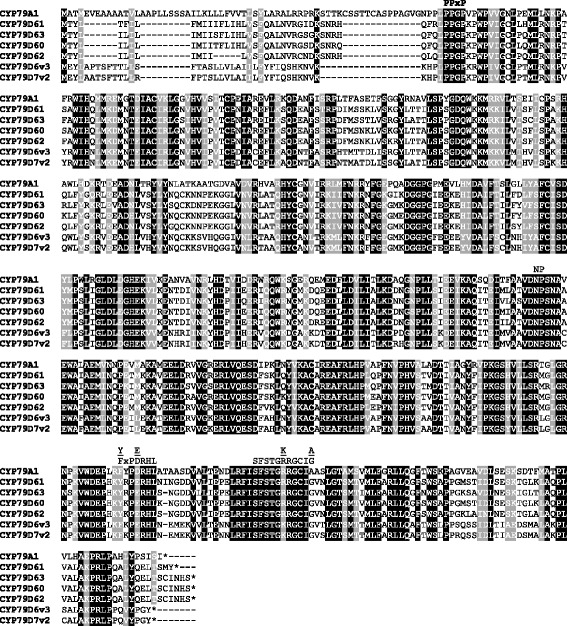
Amino acid sequence alignment of *Erythroxylum* CYP79s with CYP79A1 from *Sorghum bicolor* and CYP79D6v3 and CYP79D7v2 from *Populus trichocarpa*. Black boxes mark conserved residues and grey boxes mark residues with similar physicochemical properties. The conserved motifs are labeled and ‘NP’ indicates the exchange of the generally conserved CYP motif, Thr-(Thr/Ser), with the Asn-Pro motif typical of the CYP79 family

**Fig. 2:**
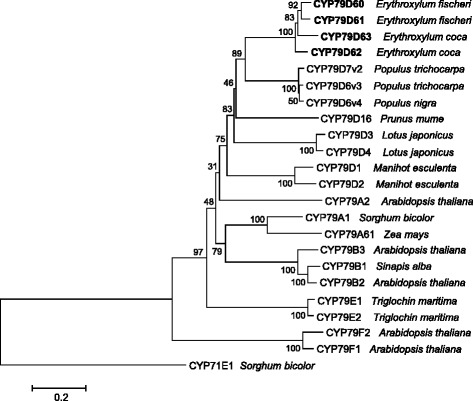
Rooted phylogenetic tree of *Erythroxylum* CYP79D proteins and characterized CYP79 proteins from other plants. The tree was inferred by using the neighbor joining method and *n* = 1000 replicates for bootstrapping. Bootstrap values are shown next to each node. CYP71E1 was used as the outgroup. The tree is drawn to scale, with branch lengths measured in the number of substitutions per site. Enzymes described in this study are shown in bold. Accession numbers: CYP71E1, AF029858.1; CYP79F1, NM_101507.2; CYP79F2, AF275259.1; CYP79B2, NM_120158.2; CYP79B1, AF069494.1; CYP79B3, NM_127798.3; CYP79A61, KP297890.1; CYP79A1, U32624.1; CYP79D2, AY834390.1; CYP79D1, AY834391.1; CYP79E2, AF140610.1; CYP79E1, AF140609.1; CYP79A2, AF245302.1; CYP79D4, AY599896.1; CYP79D3, AY599895.1; CYP79D16, AB920488.1; CYP79D7v2, KF562516.1; CYP79D6v3, KF562515.1; CYP79D6v4, KF870998.1

Motifs reported to be conserved in nearly all P450 enzymes, such as the ProProxxPro motif at the N-terminus, the heme binding site ProPheGlyxGlyArgArgxCysxGly, and the ProGluArgPhe motif, could be identified in the obtained *Erythroxylum* CYP79 sequences (Fig. 1). In comparison to the general P450 consensus sequences [26], *Erythroxylum* CYP79 motifs showed substitutions characteristic for the CYP79 family. Moreover, a CYP79-specific AsnPro motif in one of the proposed substrate binding sites [26] was also found in the *Erythroxylum* sequences (Fig. 1). A dendrogram analysis showed that *Erythroxylum* CYP79 enzymes grouped together with CYP79D6v3, CYP79D7v2, and CYP79D6v4 from poplar and CYP79D enzymes from other plants (Fig. 2).

**Fig. 3:**
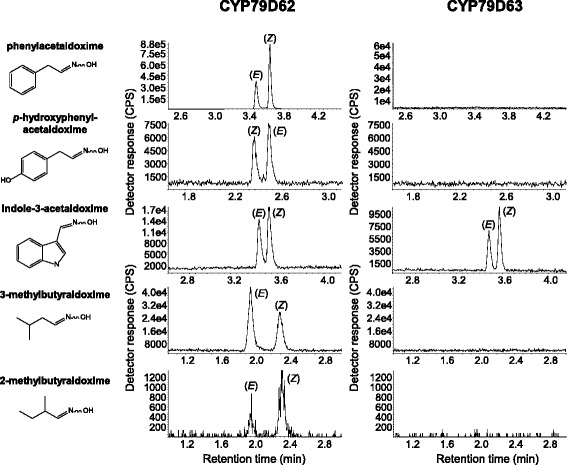
Biochemical characterization of *Erythroxylum coca* CYP79D62 and CYP79D63. The genes were heterologously expressed in *Saccharomyces cerevisae* and microsome preparations containing the recombinant proteins were incubated with the potential amino acid substrates L-Phe, L-Tyr, L-Trp, L-Leu, and L-Ile. The respective reaction products of each substrate are depicted sequentially next to their LC-MS/MS traces

**Fig. 4:**
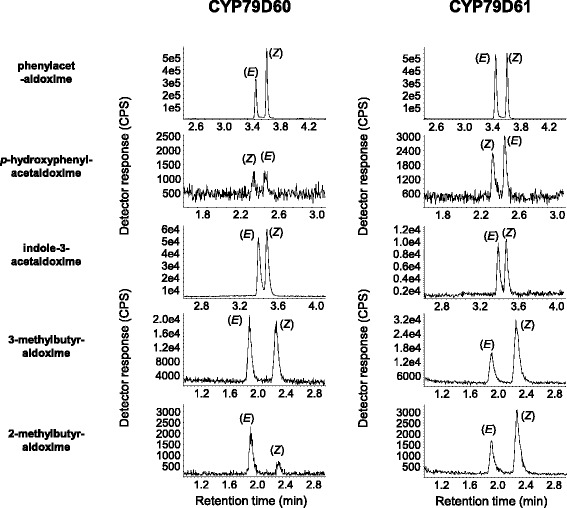
Biochemical characterization of *Erythroxylum fischeri* CYP79D60 and CYP79D61. The genes were heterologously expressed in *Saccharomyces cerevisae* and microsome preparations containing the recombinant proteins were incubated with the potential amino acid substrates L-Phe, L-Tyr, L-Trp, L-Leu, and L-Ile. The names of the respective reaction products are listed sequentially next to their LC-MS/MS traces

To test the enzymatic activity of the identified *Erythroxylum* CYP79s, genes were heterologously expressed in *Saccharomyces cerevisiae* (WAT_11_) and microsomes harbouring recombinant protein were incubated with the potential amino acid substrates L-phenylalanine, L-tyrosine, L-tryptophan, L-leucine, and L-isoleucine in the presence of NADPH as cosubstrate. CYP79D63 showed narrow substrate specificity and was only able to accept tryptophan as substrate, converting it into (*E/Z*)-indole-3-acetaldoxime (Fig. 3). In contrast, CYP79D62, CYP79D60, and CYP79D61 accepted all tested amino acids and produced (*E/Z*)-phenylacetaldoxime, (*E/Z*)-*p*-hydroxyphenylacetaldoxime, (*E/Z*)-indole-3-acetaldoxime, (*E/Z*)-3-methylbutyraldoxime, and (*E*/*Z*)-2-methylbutyraldoxime, respectively, from the amino acids listed above (Figs. 3 and 4). Assays using microsomes from yeast cells expressing the empty vector, assays without NADPH and assays with boiled proteins showed no activity (data not shown).

*K*_m_ values for the different substrates of CYP79D60, CYP79D62, and CYP79D63 are given in Table 2. Since measurements of carbon monoxide difference spectra failed, we were not able to determine the protein concentrations in the microsomes and thus to calculate the turnover numbers for the different substrates. Instead, the relative product formation with 1 mM of the respective amino acid substrate was measured (Table 2). For CYP79D60 and CYP79D62, the combination of relatively low *K*_m_ values for L-Phe and L-Leu combined with a high rate of product formation suggest that these amino acids are the preferred substrates in planta. Although CYP79D63, which only accepted L-Trp as a substrate, had a lower *K*_m_ value for this amino acid than CYP79D60 and CYP79D61 (0.48 ± 0.05 mM versus 2.74 ± 0.11 mM and 1.09 ± 0.04 mM, respectively), the rate of product formation indicates a low turn-over number for this enzyme.

**Table 2:** Kinetic parameters for CYP79D60, CYP79D62, and CYP79D63. The maximal velocities were measured in the presence of 1 mM substrate. CYP79D63 showed no activity with L-Phe, L-Leu, L-Ile, and L-Tyr

	CYP79D60		CYP79D62		CYP79D63	
substrate	*K*_m_ (mM)	Maximal velocity (ng*h^-1^*assay^-1^)	*K*_m_ (mM)	Maximal velocity (ng*h^-1^*assay^-1^)	*K*_m_ (mM)	Maximal velocity (ng*h^-1^*assay^-1^)
L-Phe	0.58 ± 0.05	160.44 ± 4.33	0.67 ± 0.07	424.00 ± 15.24	-	-
L-Leu	0.23 ± 0.08	68.58 ± 2.49	0.59 ± 0.08	256.90 ± 7.45	-	-
L-Ile	1.28 ± 0.25	32.62 ± 5.16	3.27 ± 0.42	42.55 ± 3.49	-	-
L-Trp	2.74 ± 0.11	26.50 ± 0.32	1.09 ± 0.04	16.36 ± 0.31	0.48 ± 0.05	2.92 ± 0.14
L-Tyr	6.09 ± 1.32	4.66 ± 0.43	4.99 ± 0.46	73.18 ± 1.93	-	-

### Gene expression analysis of *Erythroxylum CYP79* genes

**Fig. 5:**
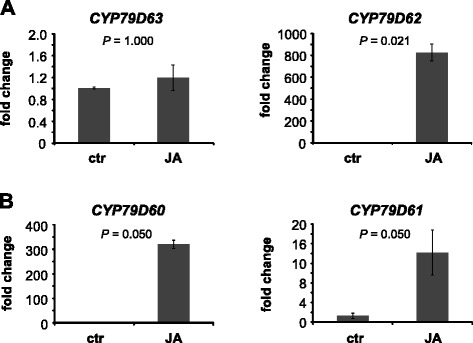
Trancript abundance of *CYP79D* genes in jasmonic acid-treated and untreated control leaves of *Erythroxylum coca* (**a**) and *E. fischeri* (**b**). Twigs were cut and placed in either tap water (ctr) or jasmonic acid (200 μM) for 18 h. Gene expression was determined by qRT-PCR. Means and standard errors are shown (*E. coca*, *n* = 4; *E. fischeri*, *n* = 3). The Kruskal-Wallis rank sum test was used to test for statistical significance. *P*-values ≤ 0.05 indicate significant difference between the treatments. ctr, control treatment; JA, jasmonic acid treatment

Quantitative real-time PCR (qRT-PCR) was used to compare transcript accumulation of *CYP79* genes between untreated and JA-treated twigs in *E. coca* and *E. fischeri*. To identify reference genes with stable expression under our experimental conditions, we analysed transcript accumulation of a set of nine potential *E. coca* qRT-PCR reference genes [27] in untreated and JA-treated leaves of *E. coca* and *E. fischeri* (Additional file 1: Tables S1 and S2). Expressed protein Ec6409 and the clathrin adaptor complex subunit Ec11142 were chosen as reference genes for qRT-PCR analysis of *CYP79* genes in *E. coca* and *E. fischeri*, respectively, based on their low Ct value variability between the different treatments (Additional file 1: Tables S1 and S2). In *E. coca*, *CYP79D62* showed a significantly upregulated gene expression in JA-treated twigs in comparison to untreated controls (Fig. 5a). In contrast, transcript accumulation of *CYP79D63* was not influenced by the treatment (Fig. 5a). In *E. fischeri*, *CYP79D60* and *CYP79D61* were both significantly upregulated after JA treatment (Fig. 5b), but the average Cq value for *CYP79D61* was higher than the average Cq value for *CYP79D60* in JA-treated leaves (27.4 versus 20.5), suggesting higher gene expression for *CYP79D60* in comparison to *CYP79D61* after JA treatment.

### Accumulation of aldoximes, indole-3-acetic acid, and amino acids in JA-treated *Erythroxylum* plants

**Fig. 6:**
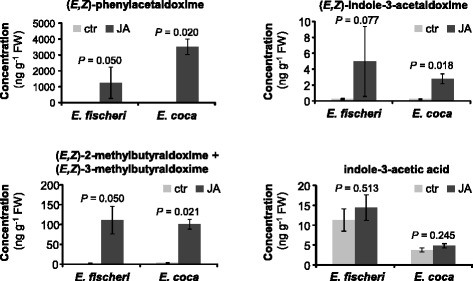
The accumulation of different aldoximes and indole-3-acetic acid (IAA) in jasmonic acid-treated and untreated control leaves of *Erythroxylum coca* and *E. fischeri*. Twigs were cut and placed in either tap water (ctr) or jasmonic acid (200 μM) for 18 h. Aldoximes and IAA were extracted with methanol and analyzed using LC-MS/MS. Means and standard errors are shown (*E. coca*, *n* = 4; *E. fischeri*, *n* = 3). The Kruskal-Wallis rank sum test was used to test for statistical significance. *P*-values ≤ 0.05 indicate significant difference between the treatments. ctr, control treatment; JA, jasmonic acid treatment

To test whether CYP79 products accumulate in JA-treated and untreated leaves of *E. coca* and *E. fischeri,* we analysed leaf methanol extracts using liquid chromatography-tandem mass spectrometry. (*E/Z*)-Phenylacetaldoxime, (*E/Z*)-indole-3-acetaldoxime, (*E/Z*)-2-methylbutyraldoxime, and (*E/Z*)-3-methylbutyraldoxime showed significantly increased accumulation in both species after JA-treatment in comparison to untreated controls (Fig. 6). Only trace amounts of these aldoximes could be detected in untreated leaves. Notably, the induced accumulation of (*E/Z*)-2-methylbutyraldoxime and (*E/Z*)-3-methylbutyraldoxime corresponded well with the emission of these compounds from JA-treated leaves (Table 1). The absence of the aromatic aldoximes (*E/Z*)-phenylacetaldoxime and (*E/Z*)-indole-3-acetaldoxime in the volatile blends (Table 1) is most likely due to their low volatility in comparison to the aliphatic aldoximes. In contrast to the aldoximes, indole-3-acetic acid (IAA), a potential conversion product of (*E/Z*)-indole-3-acetaldoxime, was constitutively produced in untreated and JA-treated leaves of both *E. coca* and *E. fischeri* (Fig. 6).

The analysis of amino acids as potential CYP79 substrates in JA-treated leaves vs. untreated control leaves revealed a significant induction for L-Ala, L-Val, L-Thr, L-Leu, L-Ile, L-His, L-Phe, L-Trp, and L-Tyr in *E. coca* and for L-Ala, L-Asp, and L-Gln in *E. fischeri* (Additional file 1: Table S3).

## Discussion

The production of volatiles in response to insect herbivory appears to be a widespread part of plant defence. Herbivore-induced volatiles can influence the feeding or oviposition behaviour of herbivores and are described to attract herbivore enemies such as parasitic wasps, predatory arthropods, and insectivorous birds [1]. Jasmonic acid, a phytohormone known to be involved in several physiological processes, plays an important role in triggering different plant defence reactions including volatile formation [23, 28, 29]. Thus, pure JA or its derivatives and mimics are often used as artificial elicitors for the induction of vegetative volatile emission [22, 30, 31]. In this study we showed that JA also induced the emission of complex volatile blends in *E. coca* and *E. fischeri*. The blends were dominated by terpenes and GLVs, but also possessed nitrogen-containing compounds such as the nitrile benzyl cyanide, phenylnitroethane, and some aliphatic aldoximes (Table 1). The roles of herbivore-induced nitrogenous volatiles in direct and indirect plant defense have recently been investigated in poplar. Olfactometer experiments showed that benzyl cyanide and two other volatile nitriles had a strong repellant activity against gypsy moth caterpillars, a generalist herbivore known to feed on poplar [13]. Volatile aliphatic aldoximes were found to be attractive for a parasitoid of gypsy moth larvae in laboratory as well as field experiments [12], and the semi-volatile (*E,Z*)-phenylacetaldoxime, which accumulated after herbivory in poplar leaves, decreased survival and weight gain of gypsy moth larvae in feeding experiments [11]. Since in the present study volatile aliphatic and aromatic aldoximes, nitriles and nitro compounds and the semi volatile (*E,Z*)-phenylacetaldoxime were found to be emitted from or accumulated in the two investigated *Erythroxylum* species after treatment with JA in the same order of magnitude as that previously reported for herbivore-damaged poplar leaves [11–13], these compounds might play similar roles in plant defence against natural *Erythroxylum* herbivores such as *Eloria noyesi* and *Eucleodora cocae*, two caterpillar pests, or the leaf cutting ant *Acromyrmex spp*. [32].

Using homology-based searches, four genes with similarity to *CYP79*s from other plants could be identified in *E. coca* and *E. fischeri. CYP79D60* and *CYP79D61* from *E. fischeri* and *CYP79D62* from *E. coca* were significantly upregulated after JA treatment (Fig. 4) and the encoded enzymes had broad substrate specificity (Figs. 2 and 3). The kinetic parameters of CYP79D60 and CYP79D62 were in the range reported for those of previously characterized poplar CYP79 enzymes [11]. Although the *K*_m_ values were relatively high, it has been suggested that the low substrate affinity of CYP79 enzymes has evolved to avoid possible depletion of free amino acid pools in plants [19]. Considering both the *K*_m_ and maximal velocity values for the conversion of the different substrates (Table 2), it is likely that CYP79D60 and CYP79D62 accept L-phenylalanine, L-leucine, L-isoleucine, and L-tryptophan as substrates *in planta*. Moreover, the accumulation and emission of their aldoxime products after JA treatment (Table 1; Fig. 5) coupled with the JA-induced expression of their genes (Fig. 4) indicate that CYP79D62 and CYP79D60 contribute to herbivore-induced aldoxime formation in *E. coca* and *E. fischeri*, respectively. The JA-induced production of aldoximes might be further promoted by the increased accumulation of the respective amino acid substrates in JA-treated leaves (Additional file 1: Table S1). Since CYP79D60 and CYP79D61 are highly similar to each other (93 % amino acid identity; Fig. 1) and showed no remarkable differences in *in vitro* assays (Fig. 3), the kinetic parameters of CYP79D61 were not determined in this study. Although it is likely that CYP79D61 has similar kinetic constants to CYP79D60, the lower expression level of *CYP79D61* in JA-treated leaves in comparison to *CYP79D60* suggests only a minor role for this enzyme in aldoxime production in *E. fischeri*.

While CYP79D60, CYP79D61, and CYP79D62 are likely involved in plant defense, the biological function of CYP79D63 remains unclear. In contrast to the other three enzymes, CYP79D63 accepted exclusively L-tryptophan as substrate (Fig. 2). The affinity of CYP79D63 for L-tryptophan was higher in comparison to CYP79D60, CYP79D61, and CYP79D62 (Table 2); however, the low relative product formation indicates a low turnover number for this enzyme. Since gene expression was not influenced by JA treatment, it is unlikely that CYP79D63 contributes to herbivore-induced accumulation of (*E,Z*)-indole-3-acetaldoxime. In many plants, the conversion of (*E,Z*)-indole-3-acetaldoxime into the corresponding acid is thought to serve as an alternative route for the formation of auxin [33–36] and thus it is conceivable that CYP79D63 might produce (*E,Z*)-indole-3-acetaldoxime as precursor for constitutive auxin formation in leaves or other growing plant parts of *E. coca*. A comprehensive correlation between *CYP79D63* gene expression and the accumulation of auxin in different plant organs and different developmental stages might help to elucidate the potential role of CYP79D63 in auxin formation.

As a result of domestication, many crop plants show altered levels of secondary compounds in comparison to their wild relatives [37]. *E. coca*, for example, has been cultivated for thousands of years and has been selected for high-level production of the pharmacologically active tropane alkaloid cocaine [38]. The cultivated species contains 20-100 times more cocaine in its leaves then closely related wild species [39]. While such selection for high-level production of useful compounds or for low-level production of undesired compounds is controlled by the breeder, domestication can also have unrecognized and unwanted side effects. The accumulation of an inactive allele of (*E*)-β-caryophyllene synthase during breeding of North American maize, for instance, led to the loss of (*E*)-β-caryophyllene production in most of these lines [40]. (*E*)-β-Caryophyllene is usually released as volatile from herbivore-damaged maize leaves and roots and has been shown to be involved in different indirect defence reactions above and below ground [40–43]. In this study we showed that *E. coca* and *E. fischeri* accumulate and release the same aldoximes, nitriles, and nitro compounds after JA-treatment in comparable amounts, suggesting that domestication did not alter these plant defence responses in cultivated *E. coca*. Whether the quantitative differences between other single compounds in the JA-induced volatile bouquets of *E. coca* and *E. fischeri* are species specific or are the result of the breeding of *E. coca*, is still unclear.

## Conclusions

Herbivore-induced volatile blends are in general very complex and contain dozens of substances. However, the enzymatic machinery behind this complexity is often astonishingly simple, comprising only a handful of enzymes with broad substrate and/or product specificity. Terpene synthases, the key enzymes in terpene biosynthesis, for instance, can produce mixtures of up to 50 compounds from one substrate [44]. Moreover, methyltransferases and acyltransferases involved in the formation of volatile esters have been reported to accept multiple substrates [31, 45, 46]. Such promiscuity in the substrate and/or product specificity of volatile-producing enzymes seems to be a general phenomenon that allows plants to efficiently produce a large mixture of different volatiles with only a limited number of enzymes. Mixtures may have specific advantages in plant defense [47]. Recently we showed that two poplar CYP79s involved in volatile aldoxime formation also exhibit broad substrate specificity in contrast to all other previously described CYP79s [11]. The *Erythroxylum* enzymes characterized in this study represent the second example for CYP79s having broad substrate specificity and it is thus tempting to speculate that such promiscuity might be a general feature for CYP79s forming herbivore-induced volatiles. However, further research on volatile aldoxime-producing CYP79 enzymes from diverse plant families is still needed to substantiate this assertion and to understand the evolutionary and structural causes of broad substrate specificity in this enzyme class.

## Methods

### Plant material and plant treatment

Seeds of *Erythroxylum coca* var *coca* were obtained from the botanical garden Bonn, Germany, and were germinated in sterilized potting soil. Live plants of *E. fischeri* were collected in Kenya and shipped to the MPICE. Plants were grown in a growth chamber set at 22 °C under a 12 h/12 h light/dark cycle, with humidity of 65 % and 70 %, respectively, and were fertilized once a week with Ferty 3 (15-10-15) and Wuxal Top N (Planta Düngemittel, Regenstauf, Germany). The cultivation of *E. coca* was authorized by the Bundesinstitut für Arzneimittel und Medizinprodukte (BfArM) (permit number, BtM 4515971).

For jasmonic acid (JA) treatment, JA (100 mg/ml ethanol) was diluted in tap water to a final concentration of 200 μM. Ethanol was diluted in tap water in the same way and used as control. From each *E. coca* plant (*n* = 4), four twigs of about 15–20 cm in length were cut and immediately placed in glass beakers containing either JA (two twigs) or control solution (the other two twigs). For *E. fischeri*, two twigs of about 20 cm in length were cut from each plant (*n* = 3) and only one twig was used per treatment. Twigs were left in JA or control solution overnight for 18 h before the volatile collection.

### Volatile collection and analysis

Volatile collections were performed in a growth chamber under conditions as described above. Glass beakers containing the *Erythroxylum* twigs were separately placed in 3 l glass desiccators which were tightly closed. Purified air pumped into the desiccator at a rate of 0.5 l min^-1^ came into contact with the plant and left the vessel through a filter packed with 30 mg Super-Q (ARS, Inc., Gainesville, FL, USA). Volatiles were collected for 5 h (9 am–2 pm). After the collection, the plant material was immediately frozen in liquid nitrogen for further analysis. The volatile compounds were desorbed from the filters by eluting the filter twice with 100 μl dichloromethane containing nonyl acetate as an internal standard (10 ng μl^-1^).

Qualitative and quantitative volatile analysis was conducted using an Agilent 6890 Series gas chromatograph (Agilent Technologies GmbH, Waldbronn, Germany) coupled to an Agilent 5973 quadrupole mass selective detector (interface temp, 270 °C; quadrupole temp, 150 °C; source temp, 230 °C; electron energy, 70 eV) or a flame ionization detector (FID) operated at 300 °C, respectively. The constituents of the volatile bouquet were separated using a ZB-WAX column (Phenomenex, Aschaffenburg, Germany, 60 m × 0.25 mm × 0.15 μm) and He (MS) or H_2_ (FID) as carrier gas. The sample (1 μL) was injected without split at an initial oven temperature of 40 °C. The temperature was held for 2 min and then increased to 225 °C with a gradient of 5 °C min^-1^, held for another 2 min, and then further increased to 250 °C with 100 °C min^-1^ and a hold for 1 min.

Compounds were identified by comparison of retention times and mass spectra to those of authentic standards obtained from Fluka (Seelze, Germany), Roth (Karlsruhe, Germany), Sigma (St. Louis, MO, USA), and Bedoukian (Danbury, CT, USA) or by reference spectra in the Wiley and National Institute of Standards and Technology libraries. The absolute amount of all compounds was determined based on their FID peak area in relation to the area of the internal standard.

### Plant tissue sampling, RNA extraction, and reverse transcription

*Erythroxylum* leaf material was harvested immediately after the volatile collection, flash-frozen with liquid nitrogen, and stored at -80 °C until further processing. After grinding the frozen leaf material in liquid nitrogen to a fine powder, total RNA was isolated using an InviTrap Spin Plant RNA kit (Stratec, Berlin, Germany) according to manufacturer’s instructions. RNA concentration, purity, and quality were assessed using a spectrophotometer (NanoDrop 2000c, Thermo Scientific, Wilmington, DE, USA) and an Agilent 2100 Bioanalyzer. RNA was treated with TurboDNase (ThermoFisher Scientific, https://www.thermofisher.com) prior to cDNA synthesis. Single-stranded cDNA was prepared from 1 μg of DNase-treated RNA using SuperScript^TM^ III reverse transcriptase and oligo (dT_12-18_) primers (Invitrogen, Carlsbad, CA, USA).

### Identification and heterologous expression of *CYP79* genes

A TBLASTN search against an in-house 454 cDNA sequencing database of *E. coca* young leaf tissue with CYP79D6v3 from *Populus trichocarpa* (GenBank AHF20912.1) as input sequence revealed one sequence with similarity to plant CYP79s. The full-length gene was designated as *CYP79D62* according to the general P450 nomenclature (D.R. Nelson, P450 Nomenclature Committee) and could be amplified from cDNA attained from JA-treated leaves of *E. coca.* The PCR product was cloned into the sequencing vector pCR^®-^Blunt II-TOPO^®^ (Invitrogen) and both strands were fully sequenced using the Sanger method. Sequencing of several clones revealed a second *CYP79* gene that was designated as *CYP79D63*. Using the primers designed for amplification of *E. coca CYP79* genes, a *CYP79* sequence could be amplified from cDNA made from JA-treated *E. fischeri* leaves (*CYP79D60*). To identify further potential *CYP79D* candidates, primers specific to conserved regions among the obtained genes were designed and PCR was performed with cDNA made from JA-treated *Erythroxylum* leaves. While most of the resulting amplicons were identical to *CYP79D62, CYP79D63,* and *CYP79D60*, one fragment amplified from *E. fischeri* cDNA showed sequence divergence. RacePCR was performed to obtain the full-length clone, which was designated as *CYP79D61*. Primer sequence information is given in Additional file 1: Table S4.

For heterologous expression in *Saccharomyces cerevisiae*, the complete open reading frames of *CYP79D62*, *CYP79D63*, *CYP79D61*, and *CYP79D60* were cloned into the pESC-Leu2d vector [48] as *No*tI/*Sac*I fragments. The resulting constructs were transferred into the *S. cerevisiae* strain WAT11 [49] and single yeast colonies were picked to inoculate starting cultures containing 30 mL SC minimal medium lacking leucine (6.7 g L^-1^ yeast nitrogen base without amino acids, but with ammonium sulfate). Other components: 100 mg L^-1^ of L-adenine, L-arginine, L-cysteine, L-lysine, L-threonine, L-tryptophan and uracil; 50 mg L^-1^ of the amino acids L-aspartic acid, L-histidine, L-isoleucine, L-methionine, L-phenylalanine, L-proline, L-serine, L-tyrosine, L-valine; 20 g L^-1^ D-glucose. The cultures were grown overnight at 28 °C and 180 rpm. One OD of the starting cultures (approx. 2 × 10^7^ cells mL^-1^) was used to inoculate 100 mL YPGA full medium cultures (10 g L^-1^ yeast extract, 20 g L^-1^ bactopeptone, 74 mg L^-1^ adenine hemisulfate, 20 g L^-1^ D-glucose) which were grown for 32–35 h (until OD about 5), induced by the addition of galactose and cultured for another 15–18 h. The cultures were centrifuged (7500*g*, 10 min, 4 °C), the supernatant was decanted, and the cell pellets were resuspended in 30 mL TEK buffer (50 mM Tris-HCl pH 7.5, 1 mM EDTA, 100 mM KCl) and centrifuged again. Then, the pellets were carefully resuspended in 2 mL of TES buffer (50 mM Tris-HCl pH 7.5, 1 mM EDTA, 600 mM sorbitol, 10 g L^-1^ bovine serum fraction V protein and 1.5 mM β-mercaptoethanol) and transferred to a 50 mL conical tube. Glass beads (0.45–0.50 mm diameter, Sigma-Aldrich Chemicals, Steinheim, Germany) were added so that they filled the full volume of the cell suspension. Yeast cell walls were disrupted by 5 cycles of 1 min shaking by hand and subsequent cooling down on ice for 1 min. The crude extracts were recovered by washing the glass beads 4 times with 5 mL TES. The combined washing fractions were centrifuged (7500*g*, 10 min, 4 °C), and the supernatant was transferred to another tube and centrifuged again (100,000*g*, 60 min, 4 °C). The resulting microsomal protein fractions were homogenized in 2 mL TEG buffer (50 mM Tris-HCl, 1 mM EDTA, 30 % w/v glycerol) using a glass homogenizer (Potter-Elvehjem, Fisher Scientific, Schwerte, Germany). Aliquots were stored at -20 °C.

### Analysis of recombinant CYP79

To determine the substrate specificity of *Erythroxylum* CYP79 enzymes, yeast microsomes harboring recombinant protein were incubated for 30 min at 25 °C and 300 rpm individually with the potential substrates L-Phe, L-Val, L-Leu, L-Ile, L-Tyr, and L-Trp in glass vials containing 300 μL of the reaction mixture (75 mM sodium phosphate buffer (pH 7.0), 1 mM substrate (concentration was variable for K_*m*_ determination), 1 mM NADPH, and 10 μL of the prepared microsomes). Reaction products were analyzed using LC-MS/MS as described below.

For the determination of the K_*m*_ values, assays were carried out in triplicate and stopped by placing the samples on ice after 300 μL MeOH were added. Enzyme concentrations and incubation times were chosen so that the reaction velocity was linear during the incubation time period.

### qRT-PCR analysis

cDNA was prepared as described above and diluted 1:10 with water. For the amplification of *CYP79D* gene fragments with a length of about 100–150 bp, primer pairs were designed having a Tm ≥ 60 °C, a GC content between 40–55 %, and a primer length in the range of 20–25 nt (see Additional file 1: Table S4 for primer information). Primer specificity was confirmed by agarose gel electrophoresis, melting curve analysis, and standard curve analysis and by sequence verification of cloned PCR amplicons. Primer pair efficiency was determined using the standard curve method with fivefold serial dilution of cDNA and was found to be between 97 and 104 %. Samples were run in triplicate using the Brilliant^®^ III SYBR^®^ Green QPCR Master Mix (Stratagene, Carlsbad, CA, USA). The following PCR conditions were applied for all reactions: Initial incubation at 95 °C for 3 min followed by 40 cycles of amplification (95 °C for 5 s, 60 °C for 10 s). Plate reads were taken during the annealing and the extension steps of each cycle. Data for the melting curves were recorded at the end of cycling from 60 °C to 95 °C.

All samples were run on the same PCR machine (Bio–Rad CFX Manager 3.1, Bio-Rad Laboratory, Hercules, CA, USA) in an optical 96-well plate. Three (*E. fischeri*) or four (*E. coca*) biological replicates were analyzed as triplicates in the qRT-PCR for each of the three treatments.

### LC-MS/MS analysis of aldoximes, amino acids, and auxin

For determining amino acid and aldoxime concentration, 100 mg of plant powder was extracted with 1 mL MeOH. For the measurement of amino acids, the MeOH extract was diluted 1:10 with water and spiked with ^13^C, ^15^N labeled amino acids (algal amino acids ^13^C,^15^N, Isotec, Miamisburg, OH, USA) at a concentration of 10 μg of the mix per mL. Amino acids in the diluted MeOH extract were directly analyzed by LC-MS/MS as recently described [11].

Aldoximes were measured from MeOH extracts using an Agilent 1200 HPLC system coupled to an API 5000 tandem mass spectrometer (Applied Biosystems, Darmstadt, Germany). Formic acid (0.2 %) in water and acetonitrile were employed as mobile phases A and B, respectively, on a Zorbax Eclipse XDB-C18 column (50 × 4.6 mm, 1.8 μm, Agilent Technologies). The elution profile was: 0–4 min, 10–70 % B; 4–4.1 min, 70–100 % B; 4.1–5 min 100 % B and 5.1–7 min 10 % B at a flow rate of 1.1 mL min^–1^. The API 5000 tandem mass spectrometer was operated in positive ionization mode (ionspray voltage, 5500 eV; turbo gas temp, 700 °C; nebulizing gas, 60 psi; curtain gas, 30 psi; heating gas, 50 psi; collision gas, 6 psi). MRM was used to monitor precursor ion → product ion reactions for each analyte as follows: *m/z* 136.0 → 119.0 (collision energy (CE), 17 V; declustering potential (DP), 56 V) for phenylacetaldoxime; *m/z* 102.0 → 69.0 (CE, 13 V; DP, 31 V) for 2-methylbutyraldoxime; *m/z* 102.0 → 46.0 (CE, 15 V; DP, 31 V) for 3-methylbutyraldoxime; *m/z* 175.0 → 158.0 (CE, 17 V; DP, 56 V) for indole-3-acetaldoxime and *m/z* 152.0 → 107.0 (CE, 27 V; DP, 100 V) for *p*-hydroxyphenylacetaldoxime. The concentration of aldoximes was determined using external standard curves made with authentic standards synthesized as described in the literature [11].

Indole-3-acetic acid (IAA) was analyzed as follows: 100 mg of plant powder were extracted with 300 μL MeOH, and 200 μL of the extract was diluted 1:10 with water containing 0.1 % formic acid and loaded onto equilibrated Chromabond® HR-X polypropylene columns (45 μm, Macherey Nagel, Düren, Germany). The columns were washed with acidified water. The fraction containing the auxins was eluted with 1 mL acetonitrile, which was then dried under a stream of nitrogen gas. The samples were redissolved in 30 μL MeOH and subsequently analyzed by the same LC-MS/MS system as described above. Separations were performed on an Agilent XDB-C18 column (50 mm × 4.6, 1.8 μm). Eluents A and B were water containing 0.05 % formic acid and acetonitrile, respectively. The elution profile was: 0–0.5 min, 5 % B in A; 0.5–4.0 min, 5–50 % B; 4.1–4.5 min 100 % B and 4.6–7 min 5 % B. The flow rate was set to 1.1 mL min^-1^. The API 5000 tandem mass spectrometer was operated in positive ionization mode (ion spray voltage, 5500 eV; turbo gas temp, 700 °C; nebulizing gas, 60 psi; curtain gas, 30 psi; heating gas, 50 psi; collision gas, 6 psi). The MRM transition and parameter settings for IAA were as follows: *m/z* 176 → 130 (CE, 19 V; DP, 31 V). IAA concentration was determined by spiking the plant extracts with known amounts of ^2^H_5_-IAA (OlChemIm Ltd., Olomouc, Czech Republic).

### Sequence analysis and phylogenetic tree construction

An alignment of *Erythroxylum CYP79* genes and characterized *CYP79* genes from other plants was constructed using the MUSCLE (codon) algorithm (gap open, -2.9; gap extend, 0; hydrophobicity multiplier, 1.5; clustering method, upgmb) implemented in MEGA6 [50]. Based on the translated MUSCLE codon alignment, a tree was reconstructed with MEGA6 using a neighbor joining algorithm (model/method, JTT model; substitutions type, amino acids; rates among sites, uniform rates; gaps/missing data treatment, partial deletion; site coverage cutoff, 80 %). A bootstrap resampling analysis with 1000 replicates was performed to evaluate the tree topology.

### Statistical analysis

Differences in gene expression, volatile emission, and the accumulation of aldoximes, auxin, and amino acids between jasmonic acid-induced and untreated control plants were analyzed with Kruskal-Wallis rank sum tests for *E. coca* and *E. fischeri* separately in R version 3.1.1 [51].

### Accession numbers

Sequence data for genes in this article can be found in the GenBank under the following identifiers: CYP79D60 (KX344462), CYP79D61 (KX344460), CYP79D62 (KX344463), CYP79D63 (KX344461).

## Additional file

Additional file 1: Table S1. Expression levels of potential house-keeping genes in jasmonic acid-treated (JA) and untreated control (ctr) leaves of *Erythroxylum fischeri*. **Table S2.** Expression levels of potential house-keeping genes in jasmonic acid-treated (JA) and untreated control (ctr) leaves of *Erythroxylum coca*. **Table S3.** Amino acid concentrations in untreated (control) and jasmonic acid-treated (JA treatment) leaves of *Erythroxylum coca* and *E. fischeri*. **Table S4.** Oligonucleotides used for isolation and qRT-PCR analysis of *Erythroxylum coca* and *E. fischeri CYP79D* genes. (DOCX 38 kb)

## Abbreviations

CYP: Cytochrome P450 monooxygenase
GLV: Green leaf volatile
JA: Jasmonic acid
qRT-PCR: Quantitative real-time PCR
DMNT: (3*E*)-4,8-dimethylnona-1,3,7-triene
TMTT: (3*E*,7*E*)-4,8,12-trimethyltrideca-1,3,7,11-tetraene
