# Supplementary material for: CYP79D enzymes contribute to jasmonic acid-induced formation of aldoximes and other nitrogenous volatiles in two Erythroxylum species
Source: BMC Plant Biol. 2016 Oct 4;16:215. doi: 10.1186/s12870-016-0910-5 (PMC5050915; doi:10.1186/s12870-016-0910-5)
Supplement: Additional file 1: Table S1. — Expression levels of potential house-keeping genes in jasmonic acid-treated (JA) and untreated control (ctr) leaves of Erythroxylum fischeri. Table S2. Expression levels of potential house-keeping genes in jasmonic acid-treated (JA) and untreated control (ctr) leaves of Erythroxylum coca. Table S3. Amino acid concentrations in untreated (control) and jasmonic acid-treated (JA treatment) leaves of Erythroxylum coca and E. fischeri. Table S4. Oligonucleotides used for isolation and qRT-PCR analysis of Erythroxylum coca and E. fischeri CYP79D genes. (DOCX 38 kb) [file 12870_2016_910_MOESM1_ESM.docx]

**Additional file 1: Table S1**

Expression levels of potential house-keeping genes in jasmonic acid-treated (JA) and untreated control (ctr) leaves of *Erythroxylum fischeri*. The ΔCq values, their means, and the standard deviations (STDEV) are shown.

|  | *6409* | *10131* | *Actin* | *EF1a* | *APT2* | *11142* | *Pex4* | *Tpb* | *Pp2aa3* |
| --- | --- | --- | --- | --- | --- | --- | --- | --- | --- |
| Ef Ctr1 | 25.18 | 25.91 | 21.37 | 18.76 | 24.98 | 25.17 | 23.74 | 24.21 | 23.84 |
| Ef Ctr2 | 25.43 | 25.92 | 20.98 | 18.90 | 25.38 | 25.05 | 23.62 | 23.91 | 23.70 |
| Ef Ctr3 | 25.01 | 25.98 | 20.30 | 18.21 | 25.77 | 24.27 | 23.46 | 24.11 | 23.34 |
| Ef JA1 | 24.58 | 25.60 | 20.76 | 18.35 | 25.32 | 24.34 | 23.78 | 24.15 | 23.12 |
| Ef JA2 | 24.53 | 25.28 | 20.26 | 18.31 | 25.35 | 24.33 | 23.46 | 23.73 | 23.06 |
| Ef JA3 | 25.85 | 34.31 | 21.87 | 20.32 | 27.24 | 25.08 | 25.83 | 25.81 | 24.48 |
| **Mean (ΔCq)** | **25.10** | **27.17** | **20.92** | **18.81** | **25.67** | **24.71** | **23.98** | **24.32** | **23.59** |
| **STDEV** | **0.506** | **3.508** | **0.625** | **0.790** | **0.809** | **0.436** | **0.916** | **0.750** | **0.538** |

**Additional file 1: Table S2**

Expression levels of potential house-keeping genes in jasmonic acid-treated (JA) and untreated control (ctr) leaves of *Erythroxylum coca*. The ΔCq values, their means, and the standard deviations (STDEV) are shown.

|  | *6409* | *10131* | *Actin* | *EF1a* | *APT2* | *11142* | *Pex4* | *Tpb* | *Pp2aa3* |
| --- | --- | --- | --- | --- | --- | --- | --- | --- | --- |
| Ec Ctr1 | 25.79 | 24.88 | 21.75 | 19.02 | 25.18 | 25.17 | 24.10 | 24.07 | 24.14 |
| Ec Ctr2 | 25.56 | 25.01 | 21.33 | 18.63 | 24.19 | 25.08 | 24.00 | 24.14 | 24.07 |
| Ec Ctr3 | 25.28 | 24.43 | 21.18 | 18.23 | 23.85 | 24.88 | 23.71 | 23.88 | 23.63 |
| Ec Ctr4 | 25.51 | 25.04 | 21.40 | 18.69 | 24.36 | 25.21 | 24.33 | 24.32 | 24.09 |
| Ec JA1 | 26.12 | 25.06 | 22.00 | 19.33 | 23.91 | 25.62 | 24.95 | 24.61 | 24.21 |
| Ec JA2 | 25.41 | 24.18 | 21.58 | 18.76 | 23.22 | 25.10 | 24.17 | 24.03 | 23.92 |
| Ec JA3 | 25.33 | 23.88 | 21.21 | 18.58 | 22.93 | 24.48 | 23.94 | 23.63 | 23.64 |
| Ec JA4 | 25.54 | 25.31 | 21.60 | 19.04 | 23.09 | 25.11 | 24.39 | 24.14 | 24.13 |
| **Mean (ΔCq)** | **25.57** | **24.73** | **21.51** | **18.78** | **23.84** | **25.08** | **24.20** | **24.10** | **23.98** |
| **STDEV** | **0.272** | **0.501** | **0.280** | **0.337** | **0.752** | **0.319** | **0.372** | **0.288** | **0.227** |

**Additional file 1: Table S3**

Amino acid concentrations in untreated (control) and jasmonic acid-treated (JA treatment) leaves of *Erythroxylum coca* and *E. fischeri.* Concentrations are displayed as means ± SE in µmol g^-1^ fresh weight (*E. coca*, n = 4; *E. fischeri*, n = 3). *P*-values are based on the results from Kruskal-Wallis rank sum tests between the control and the JA treatment. *P*-values ≤ 0.05 indicate significant differences and are shown in bold.

| \|  \| ***Erythroxylum coca*** \| \| \| ***Erythroxylum fischerii*** \| \| \| \| --- \| --- \| --- \| --- \| --- \| --- \| --- \| \| **Amino acid** \| **Control**  (Mean ± SE) \| **JA treatment** (Mean ± SE) \| ***P*-value** \| **Control**  (Mean ± SE) \| **JA treatment** (Mean ± SE) \| ***P*-value** \| \| Ala \| 1.25 ± 0.1 \| 2.23 ± 0.13 \| **0.021** \| 0.99 ± 0.08 \| 1.74 ± 0.17 \| **0.050** \| \| Ser \| 1.62 ± 0.11 \| 1.51 ± 0.06 \| 0.386 \| 0.28 ± 0.03 \| 0.34 ± 0.02 \| 0.184 \| \| Pro \| 1.57 ± 0.46 \| 2.04 ± 0.27 \| 0.248 \| 4.44 ± 1.57 \| 5.83 ± 1.57 \| 0.513 \| \| Val \| 0.19 ± 0.03 \| 0.41 ± 0.03 \| **0.020** \| 0.18 ± 0.1 \| 0.34 ± 0.12 \| 0.275 \| \| Thr \| 0.34 ± 0.03 \| 0.64 ± 0.05 \| **0.021** \| 0.22 ± 0.08 \| 0.37 ± 0.04 \| 0.275 \| \| Ile \| 0.23 ± 0.04 \| 0.44 ± 0.04 \| **0.021** \| 0.19 ± 0.1 \| 0.35 ± 0.12 \| 0.376 \| \| Leu \| 0.11 ± 0.02 \| 0.22 ± 0.02 \| **0.020** \| 0.12 ± 0.07 \| 0.2 ± 0.08 \| 0.513 \| \| Asp \| 0.61 ± 0.08 \| 0.78 ± 0.08 \| 0.149 \| 0.42 ± 0.02 \| 0.73 ± 0.12 \| **0.050** \| \| Glu \| 10.22 ± 0.6 \| 9.1 ± 0.41 \| 0.149 \| 7.74 ± 0.93 \| 8.07 ± 0.99 \| 0.827 \| \| Met \| 0.04 ± 0 \| 0.04 ± 0.01 \| 0.874 \| 0.02 ± 0 \| 0.02 ± 0 \| 0.317 \| \| His \| 0.29 ± 0.04 \| 0.48 ± 0.05 \| **0.043** \| 0.2 ± 0.04 \| 0.38 ± 0.09 \| 0.127 \| \| Phe \| 0.12 ± 0.01 \| 0.27 ± 0.02 \| **0.019** \| 0.14 ± 0.02 \| 0.23 ± 0.04 \| 0.121 \| \| Arg \| 4.23 ± 0.67 \| 3.87 ± 0.34 \| 0.773 \| 6.17 ± 0.64 \| 8.01 ± 0.72 \| 0.127 \| \| Tyr \| 0.05 ± 0.01 \| 0.14 ± 0.02 \| **0.020** \| 0.03 ± 0.01 \| 0.04 ± 0.01 \| 0.261 \| \| Trp \| 0.18 ± 0.01 \| 0.34 ± 0.03 \| **0.021** \| 0.15 ± 0.02 \| 0.29 ± 0.08 \| 0.077 \| \| Asn \| 0.26 ± 0.04 \| 0.3 ± 0.05 \| 0.564 \| 0.04 ± 0 \| 0.18 ± 0.14 \| 0.637 \| \| Gln \| 2.26 ± 0.19 \| 2.76 ± 0.33 \| 0.386 \| 1.43 ± 0.37 \| 2.67 ± 0.6 \| **0.046** \| \| Lys \| 0.06 ± 0 \| 0.08 ± 0.01 \| 0.240 \| 0.08 ± 0.01 \| 0.16 ± 0.06 \| 0.077 \| |  | | | | |  | | | |  |
| --- | --- | --- | --- | --- | --- | --- | --- | --- | --- | --- | --- | --- | --- | --- | --- | --- | --- | --- | --- | --- | --- | --- | --- | --- | --- | --- | --- | --- | --- | --- | --- | --- | --- | --- | --- | --- | --- | --- | --- | --- | --- | --- | --- | --- | --- | --- | --- | --- | --- | --- | --- | --- | --- | --- | --- | --- | --- | --- | --- | --- | --- | --- | --- | --- | --- | --- | --- | --- | --- | --- | --- | --- | --- | --- | --- | --- | --- | --- | --- | --- | --- | --- | --- | --- | --- | --- | --- | --- | --- | --- | --- | --- | --- | --- | --- | --- | --- | --- | --- | --- | --- | --- | --- | --- | --- | --- | --- | --- | --- | --- | --- | --- | --- | --- | --- | --- | --- | --- | --- | --- | --- | --- | --- | --- | --- | --- | --- | --- | --- | --- | --- | --- | --- | --- | --- | --- | --- | --- | --- | --- | --- | --- | --- | --- | --- | --- | --- | --- | --- | --- |
|  |  |  |  |  |  |  |  |  |  |  |
|  |  |  |  |  |  |  |  |  |  |  |
|  |  |  |  |  |  |  |  |  |  |  |
|  |  |  |  |  |  |  |  |  |  |  |
|  |  |  |  |  |  |  |  |  |  |  |
|  |  |  |  |  |  |  |  |  |  |  |
|  |  |  |  |  |  |  |  |  |  |  |
|  |  |  |  |  |  |  |  |  |  |  |
|  |  |  |  |  |  |  |  |  |  |  |
|  |  |  |  |  |  |  |  |  |  |  |
|  |  |  |  |  |  |  |  |  |  |  |
|  |  |  |  |  |  |  |  |  |  |  |
|  |  |  |  |  |  |  |  |  |  |  |
|  |  |  |  |  |  |  |  |  |  |  |
|  |  |  |  |  |  |  |  |  |  |  |
|  |  |  |  |  |  |  |  |  |  |  |
|  |  |  |  |  |  |  |  |  |  |  |
|  |  |  |  |  |  |  |  |  |  | 0.046 |
|  |  |  |  |  |  |  |  |  |  | 0.077 |

**Additional file 1: Table S4**

Oligonucleotides used for isolation and qRT-PCR analysis of *Erythroxylum coca* and *E. fischeri* *CYP79D* genes.

| **name** | **sequence** | | **usage** |
| --- | --- | --- | --- |
| CYP79D63_qPCR_fwd1  CYP79D63_qPCR_rev1  Cyp79D62_qPCR_fwd1  Cyp79D62_qPCR_rev1a  Cyp79D60_qPCR_fwd1  Cyp79D60_qPCR_rev1/3  Cyp79D61_qPCR_fwd2  Cyp79D61_qPCR_rev2  Ec/Ef6409_qPCR_fwd2  Ec/Ef6409_qPCR_rev2  Ec/Ef11242_qPCR_fwd  Ec/Ef11242_qPCR_rev  Ec10131_Fwd  Ec10131_Rev  Ec/EfActin_Fwd2  Ec/EfActin_Rev2  Ec/EfEF1a_Fwd2  Ec/EfEF1a_Rev2  EcAPT2_Fwd  EcAPT2_Rev  Ec/EfPex4_Fwd2  Ec/EfPex4_Rev2  Ec/EfTPB1_Fwd1  Ec/EfTPB1_Rev1  EcPp2aa3_Fwd  EcPp2aa3_Rev  CYP79D60/62/63_fwd  CYP79D60/62/63_rev  CYP79D61_fwd  CYP79D61_rev  CYP79D60/62/63_fwd_*Not*I  CYP79D60/62/63_rev_*Sac*I  CYP79D61_fwd_*Not*I  CYP79D61_rev_*Sac*I  CYP79D61_search_fwd  CYP79D61_search_rev  CYP79D61_3’RACE  CYP79D61_5’RACE | | GGACAACCCATCAAATGCCG  TTCGAGTTGCGTCCAAGCCT  GGACAATCCATCAAACGCTG  CCTTGACGTATTGGAGCTTCAC  TTAGACTCCATCCGATGGAAGC  GAGCACCACATCATCACCATTTT  ACGTCACCTGAACATTAATGGTG  CTCATTAAGATCAATGGATGACTT  GATGAGCTTCGTGAAATCCCTG  CCATCCTCTGTTGCTTCAGAAC  ACATTACCAAAGCAGGCTCATACG  TACATCTTCTCACCACCAACACAGG  TGGAAGGGTAGTGGGGTAACAATG  GAGCGTAGTCGTCAGAGAAGGC  GTGTCTGGATTGGAGGGTCTATC  CTGGACCAGACTCATCATACTCAC  ATCCAACTCCAAGGATGACCC  CAATGTGTGAGGTGTGGCAATC  ACTCAGAGAGCGAGAGAGGATGTT  TCAACTCCAGCAACCACAGAAATG  CATCTGAGACTCCTTATGAGGGTG  CACTTGAGGAGGCTGTAAAGGA  CTGTATCAGGTCCAGTTCATGCAG  AGTGGAGTAGGTAGGGATAGGAG  TGCTCCTGTTATGGGTCCTGAAG  CATCTGGGTCCTCACTCAACTCCG  ATGACCTACTTGATACTCATTCTTATCATGA  CTACGAATGATTAATGCAGCTTGTC  ATGACCTACTTAACATTCATTCTTT  TTAATACATGCTTGTCAGTTCTTG  AGGCGGCCGCAATGACCTACTTGATACTCATTCTTATCATG TTGAGCTCCTACGAATGATTAATGCAGCTTGTC  TTTGCGGCCGCAATGACCTACTTAACATTCATTCTTT  GTTGAGCTCTTAATACATGCTTGTCAGTTCTTG  CCTCCTGGTCCTAAACCAT  GGCAAGCGAGGCTTT  CACCTGAACATTAATGGTGAT  GACGGCGAAAGAATCG | qRT-PCR  qRT-PCR  qRT-PCR  qRT-PCR  qRT-PCR  qRT-PCR  qRT-PCR  qRT-PCR  qRT-PCR  qRT-PCR  qRT-PCR  qRT-PCR  qRT-PCR  qRT-PCR  qRT-PCR  qRT-PCR  qRT-PCR  qRT-PCR  qRT-PCR  qRT-PCR  qRT-PCR  qRT-PCR  qRT-PCR  qRT-PCR  qRT-PCR  qRT-PCR  ORF isolation  ORF isolation  ORF isolation  ORF isolation  cloning  cloning  cloning  cloning  fragment  fragment  RACE PCR  RACE PCR |
